# Isolated 720-Degree Tubal Torsion Associated With Tubal Ectopic Pregnancy: A Case Report and Review of Previously Reported Cases

**DOI:** 10.7759/cureus.111491

**Published:** 2026-06-25

**Authors:** Bourhan Alrayes, Mohammad Hassan Alrayes, Hiba Mihyar, Bishr Shibani

**Affiliations:** 1 General Surgery, Islamic Hospital, Amman, JOR; 2 Colorectal Surgery, University Hospital of Coventry and Warwickshire, Coventry, GBR; 3 Gynecology and Obstetrics, Central Hospital of Cambrai, Cambrai, FRA; 4 Emergency Medicine, University Hospitals Birmingham NHS Foundation Trust, Birmingham, GBR; 5 Science and Technology, Nottingham Trent University, Nottingham, GBR

**Keywords:** abdominal pain, ectopic pregnancy, isolated tubal, laparoscopy, salpingectomy

## Abstract

Isolated tubal torsion (ITT) is uncommon, particularly in ectopic pregnancy. The coexistence of these conditions presents a significant diagnostic challenge and requires prompt surgical intervention. Only four cases of ITT secondary to ectopic pregnancy have been reported in the English-language literature. A 25-year-old woman (G4P2) with a history of a recent cesarean section complicated by pelvic infection presented with acute left lower abdominal pain and six weeks of amenorrhea. A clinical examination revealed left adnexal tenderness. Transvaginal ultrasonography demonstrated an 8 cm heterogeneous adnexal mass and a serum β-hCG level of 4,990 mIU/mL. Emergency laparoscopy identified an ectopic pregnancy involving approximately two-thirds of the left fallopian tube, which had undergone torsion of 720 degrees. Laparoscopic left salpingectomy was successfully performed. The patient’s postoperative course was uneventful, with a rapid decline in β-hCG levels. This case highlights the importance of considering ITT in pregnant women presenting with acute pelvic pain and adnexal masses, particularly in the presence of prior pelvic inflammatory or surgical pathology.

## Introduction

Ectopic pregnancy is a leading cause of maternal morbidity and mortality in the first trimester, with an incidence of approximately 2% of all pregnancies, most commonly occurring in the fallopian tube [1]. However, isolated tubal torsion (ITT) secondary to tubal ectopic pregnancy is exceedingly rare [2]. 

ITT normally occurs in the presence of pre-existing adnexal pathologies, such as hydrosalpinx, paraovarian cysts, or neoplasms. These conditions are considered predisposing factors for torsion [3]. An ectopic gestational sac may mechanically predispose the tube to torsion; however, no established preoperative diagnostic or therapeutic guidelines currently exist for this rare association [4]. Other predisposing factors of tubal torsion may include anatomical distortion of the pelvis due to previous surgery or pelvic inflammatory disease (PID), which may alter tubal mobility [5,6]. Abdominal pain is the most common presenting symptom of ectopic pregnancy and ITT, along with other associated symptoms such as nausea and vomiting [1,2]. Although ectopic pregnancies are diagnosed using transvaginal ultrasonography (TVUS), ITT is not regularly found on TVUS and is diagnosed preoperatively [1,2].

Early recognition is essential because ITT may result in vascular compromise progressing to ischemic necrosis and tubal perforation, often necessitating salpingectomy [2,5]. 

A literature search of PubMed was performed until March 2026 using combinations of the terms “isolated tubal torsion”, “ectopic pregnancy”, and “tubal pregnancy torsion”. We reviewed English-language case reports describing ITT associated with tubal ectopic pregnancy. The reference lists of retrieved articles were manually screened to identify additional relevant reports. 

This case is reported due to the infrequency and diagnostic challenge posed by 720-degree tubal torsion associated with ectopic pregnancy in a patient with a recent history of cesarean section and subsequent pelvic infection. This association is extremely rare, with only four previous case reports in the English literature and eight case reports in total. To our knowledge, previously published reports have not clearly documented the degree of torsion associated with tubal ectopic pregnancy. Here, we report this case to highlight the diagnostic challenge and surgical implications of this rare pathology. 

## Case presentation

A 25-year-old woman (G4P2) presented to the emergency department with a one-day history of left lower abdominal pain associated with vaginal spotting. There were no associated gastrointestinal or urinary symptoms, and the family history was unremarkable. She reported six weeks of amenorrhea. Forty-eight hours prior to presentation, the patient had attended an outpatient clinic where serum β-hCG was 4,990 mIU/mL and transabdominal ultrasonography demonstrated no intrauterine gestational sac. A repeat ultrasound had been scheduled for one week later. The patient had a spontaneous vaginal delivery five years earlier and a caesarean section one year prior to presentation, which was complicated by postoperative pelvic infection requiring prolonged oral antibiotics. She had no significant past medical history, was not on regular medications, and had no known drug allergies.

On clinical examination, the patient was hemodynamically stable and afebrile (blood pressure 110/63 mmHg, pulse 92 bpm, O_2_ saturation 98%, temperature 36.8°C) with significant tenderness and guarding localized to the left iliac fossa. Regarding laboratory tests, all results were unremarkable, with a haemoglobin level of 11.5 g/dL, and white cell count and C-reactive protein levels were within normal limits (Table 1).

**Table 1:** Laboratory tests

Test	Value	Reference range
WBC count	8.3	4-11 x 10^3 /uL
Neutrophills	54.9%	40-75%
Lymphocytes	30.8%	20-45%
Monocytes	10.7%	2-10%
Eosinophils	3.0%	1-6%
Basophils	0.6%	0-1%
Hemoglobin (HB)	11.5	M: 13-17 g/dL; F: 12.0-15.5 g/dL
Packed cell volume	33.8	M: 40-50%; F: 36-46%
Red blood cell count	4.1	M: 4.5-5.5 x 10^6/uL; F: 3.8-5.1 x 10^6/uL
Mean corpuscular volume (MCV)	82.6	80-96 fl
Mean corpuscular hemoglobin (MCH)	27.6	27-32 pg
Mean corpuscular haemoglobin concentration (MCHC)	33.4	32-35%
Platelet count	311	150-450 x 10^3/uL
Creatinine	0.76	0.7-1.2 mg/dL
Sodium	139	135-153 mmol/L
Potassium	3.8	3.5-5.3 mmol/L
C-reactive protein	21	<5 ng/mL
β-hCG	4,990	< 5 mIU/mL

**Figure 1:**
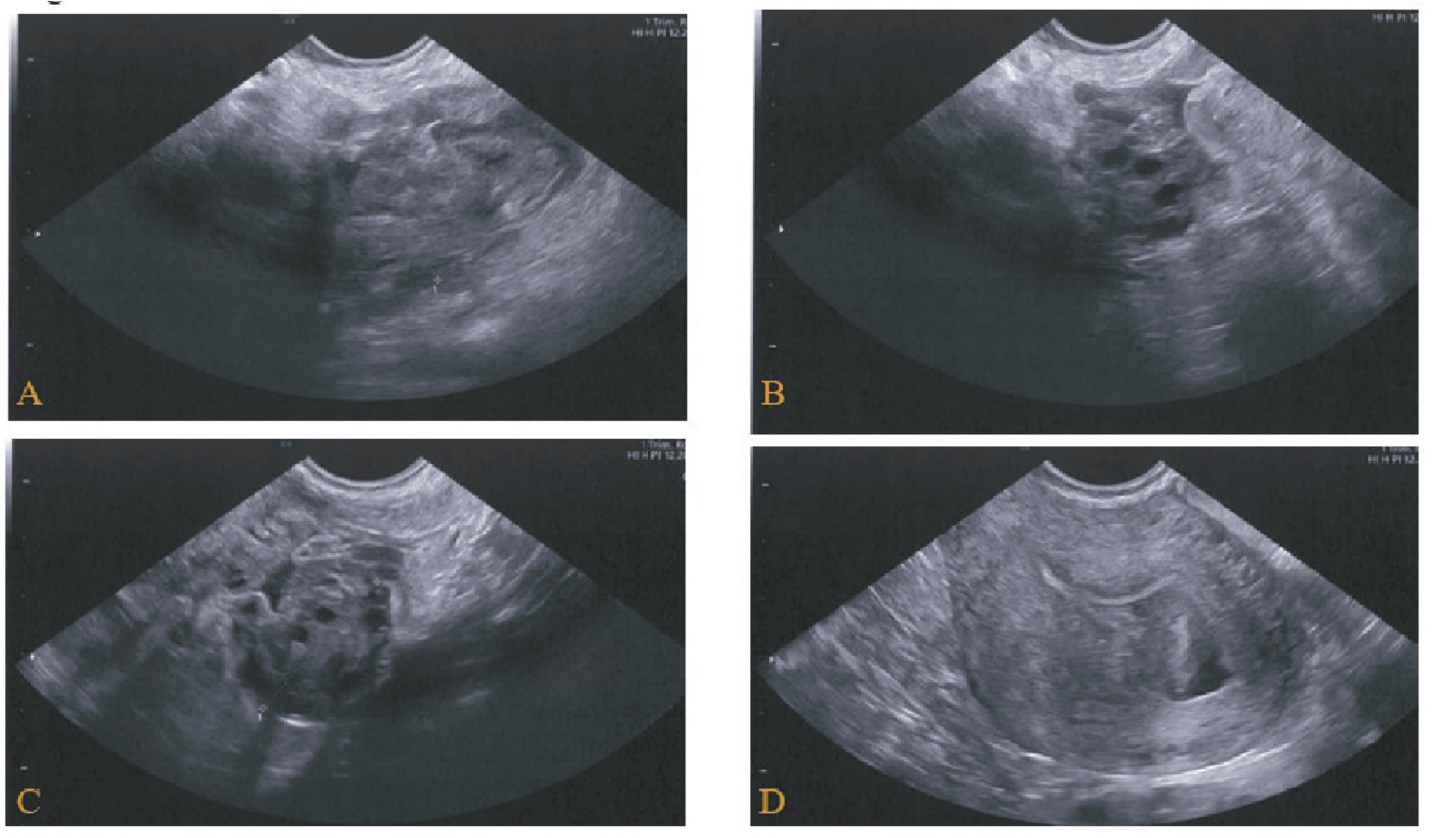
TVUS: transvaginal ultrasonography (A) a heterogeneous left adnexal mass with associated pelvic free fluid; (B,C) preserved bilateral ovarian morphology; and (D) an empty uterine cavity without evidence of intrauterine gestation.

TVUS demonstrated an empty uterine cavity with no evidence of an intrauterine gestational sac, a morphologically normal right ovary, and a prominent, heterogeneous left adnexal mass measuring 3.46 cm in its greatest dimension with free fluid in the pelvis (Figure 1).

**Figure 2:**
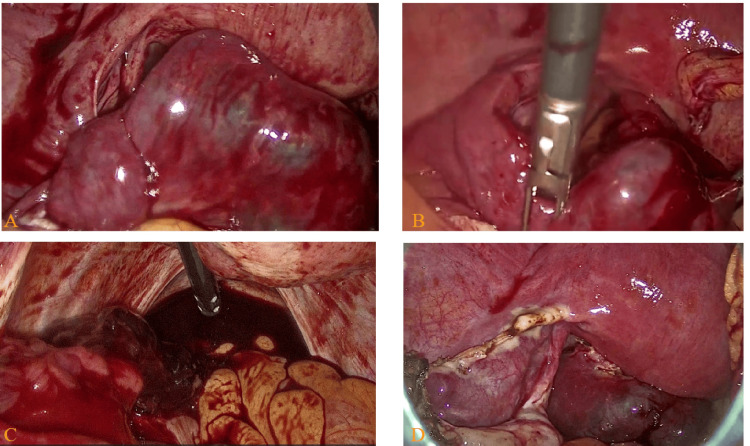
Laparoscopic intraoperative findings Laparoscopic intraoperative findings demonstrating (A) left tubal ectopic pregnancy with 720° torsion around the vascular pedicle, (B) the left fallopian tube after detorsion, which was still ischemic, (C) hemoperitoneum within the pelvis, and (D) after left salpingectomy with preservation of the left ovary.

The differential diagnosis included complicated ectopic pregnancy, complicated left ovarian cyst, haemorrhagic cyst, ovarian torsion, and, to a lesser extent, tubal torsion. As the diagnosis was uncertain, diagnostic laparoscopy under general anaesthesia was planned. The procedure was performed laparoscopically using a standard three-port technique. Intraoperative findings revealed significant pelvic adhesions, a large extra-uterine pregnancy was identified involving more than two-thirds of the left fallopian tube, and a double torsion (720 degrees) of the affected tube around its vascular pedicle (Figure 2).

Due to the extent of the tubal involvement and the ischemic changes secondary to the torsion, a left salpingectomy was performed with preservation of the left ovary. The procedure was completed without complications, and the patient’s postoperative recovery was unremarkable.

Serial monitoring of β-hCG levels post-surgery confirmed a successful resolution to normal non-pregnant levels after 48 hours. The patient reported satisfaction with the prompt diagnosis and minimally invasive surgical management and expressed reassurance following recovery at six weeks after presentation. The chronological order of events was clarified in Table 2.

**Table 2:** Chronological timeline Chronological timeline of the initial β-hCG level, presentation, transvaginal ultrasonography (TVUS), laparoscopy, and postoperative course. TVUS: transvaginal ultrasonography

Time	Event
Day -2	Outpatient review with a β-hCG level of 4990 mIU/mL
Day 0	Presented to the ED with pelvic pain and vaginal spotting
Day 0	TVUS revealed an adnexal mass and free fluid
Day 0	Emergency laparoscopy was performed
Day 2	β-hCG levels declined significantly
Postoperative follow-up at six weeks	Asymptomatic at presentation

## Discussion

To our knowledge, only four cases of isolated tubal torsion secondary to ectopic pregnancy have been reported in the English-language literature and four in the non-English literature. Full texts were accessible only for case reports in English, which are included in Table 1 for comparison. This report was limited by the small number of comparable cases reported in the literature. Our case and previously reported cases were in accordance with the age of presentation (25 vs 20-36 years), gestational age (i.e., first trimester), most had a significant obstetric history, and all were treated with salpingectomy. However, the TVUS results were inconsistent in identifying adnexal masses in previously reported cases, and none reported the degree of torsion of the fallopian tubes. In addition, half of the previously reported cases underwent laparotomy to perform a salpingectomy (Table 3).

**Table 3:** Previous English case reports reporting a tubal pregnancy with torsion of the fallopian tube found in previously reported cases.

Author	Year	Age	Obstetric history	Presentation	Imaging (TVUS)	Surgical treatment	Operative findings	Degree of torsion
Tachi et al. [12]	2023	31	G7, P2	Abdominal pain, vomiting, amenorrhea (39 days)	No uterine gestational sac, no adnexal mass, no free fluids	Diagnostic laparoscopy, left salpingectomy	Unruptured, swollen torsed left fallopian tube, hemoperitoneum	Not documented
Polat et al. [13]	2015	36	G4, P2	Abdominal pain; last menstrual period unknown	Gestational sac at the superior surface of the uterus; free fluid	Diagnostic laparoscopy was then converted to laparotomy, right salpingectomy	Unruptured mass in the right-torsed fallopian tube (75.45 mm), hemoperitoneum	Not documented
Renjit et al. [4]	2008	26	primigravida	Abdominal pain, amenorrhea (4 weeks)	No uterine gestational sac. A small corpus luteal cyst was present in the left ovary. No free fluid was apparent in the repeated TVUS.	Diagnostic laparoscopy, left salpingectomy	Unruptured mass in the left-torsed fallopian tube, hemoperitoneum	Not documented
Dicker et al. [14]	1984	20	P1, G1	Abdominal pain, amenorrhea (8 weeks)	No intrauterine gestational sac. There was a right ovarian cyst and left adnexal mass	Exploratory laparotomy, left salpingectomy, right ovarian cystectomy	Torsed left fallopian tube with hemorrhagic infarction, right dermoid cyst.	Not documented

While the coexistence of ectopic pregnancy and tubal torsion is rare, it is essential to distinguish this from other presentations of ectopic pregnancy that may similarly manifest as acute pelvic pain. Ectopic pregnancy can present as an unruptured tubal gestation, which is often managed medically; a tubal abortion, where the pregnancy is expelled into the peritoneal cavity; or a tubal rupture, which frequently presents with signs of massive hemoperitoneum and hemodynamic instability. Unlike ITT, which is characterized by mechanical twisting and vascular compromise, these common presentations generally do not involve the rotation of the tubal pedicle [1,5].

The pathophysiology of ITT in ectopic pregnancy is primarily mechanical. According to the Sellheim theory, the torsion may be precipitated by sudden changes in intra-abdominal pressure or body position, acting upon a fallopian tube that has been rendered unstable by an asymmetrical mass [6]. In this case, the 8 cm heterogeneous mass may increase the mechanical predisposition to torsion, resulting in a torsion of 720 degrees. Furthermore, the patient’s clinical history of cesarean section complicated by postoperative infection one year prior to presentation likely played a contributory role. Pelvic adhesions can create a restrictive environment or a fixed point around which the mobile portion of the tube may pivot [7]. 

Regarding diagnosis, this case underscores the limitations of ultrasound in differentiating between standard ectopic pregnancy and torsion events. While TVUS is the gold standard for identifying adnexal masses, the "whirlpool sign" typically reflects ovarian vessel twisting; this modality has limited sensitivity for ITT when ovarian morphology is preserved, often demonstrating only a nonspecific adnexal mass [8]. Consequently, the diagnosis is often made intraoperatively. The decision to perform a salpingectomy rather than detorsion in this instance was necessitated by the extent of tubal involvement, occupying more than two-thirds of the tube, and the probable compromise of tissue viability following double rotation [9]. 

Laparoscopy remains the definitive approach for the diagnosis and management of such cases. This allows for a thorough exploration of the contralateral adnexa and adhesiolysis of pre-existing adhesions that could predispose the patient to future recurrence or contralateral EPs [10,11]. The successful outcome and lack of post-operative complications in this patient demonstrate that even in the presence of complex surgical histories, prompt laparoscopic intervention remains the cornerstone of management for rare adnexal accidents. 

The strength of this report lies in its detailed intraoperative documentation and comparison with previously reported cases. Limitations include the rarity of comparable cases, the inability to establish causality between ectopic pregnancy and torsion, and the absence of preoperative radiological signs specific to isolated tubal torsion. 

## Conclusions

ITT secondary to a tubal ectopic pregnancy is extremely rare and should be considered in patients presenting with acute pelvic pain, adnexal masses, and elevated β-hCG levels. Preserved ovarian morphology on ultrasound does not exclude adnexal torsion, and tubal torsion may coexist with ectopic pregnancy despite nonspecific imaging findings. Early laparoscopic intervention remains essential for definitive diagnosis, treatment, and prevention of tubal ischemia and necrosis.
